# Anti-CD20 antibody induces the improvement of cytokine-induced killer cell activity via the STAT and MAPK/ERK signaling pathways

**DOI:** 10.3892/etm.2015.2264

**Published:** 2015-02-05

**Authors:** QI DENG, XUE BAI, HAI-RONG LV, XIA XIAO, MING-FENG ZHAO, YU-MING LI

**Affiliations:** Department of Hematology, Tianjin First Central Hospital, Tianjin 300192, P.R. China

**Keywords:** cytokine-induced killer, rituximab, human B-cell lymphoma cell lines, K562, signaling pathway, cell therapy

## Abstract

There is a current requirement for novel therapeutic strategies for the treatment of hematopoietic tumors. Residual tumor cells are the main origin of tumor relapse. The aim of this study was to eliminate the residual tumor cells of hematopoietic tumors. Cytokine-induced killer (CIK) cells are used in immunotherapy to deplete the residual cells. However, it is necessary to increase the antitumor activity and clinical applicability of CIK cells. The present study investigated the antitumor activity of CIK cells to the SU-DHL2 human B-cell lymphoma and K562 human chronic myelogenous leukemia cell lines. CD3^+^CD56^+^ cells from healthy donors were expanded in culture with cytokines and anti-CD20 monoclonal antibody (mAb; rituximab) to generate CIK cells. A preliminary investigation of their mechanism was then performed. The increase in the cytotoxicity of the CIK cells induced by the anti-CD20 mAb was associated with an increase in the expression of cytotoxic factors. The expression of components of the signal transducer and activator of transcription (STAT) and mitogen-activated protein kinase/extracellular signal-regulated kinase (MAPK/ERK) signaling pathways was found to increase. Upregulation of the expression of STAT1, STAT3 and STAT5 is important as these co-stimulatory molecules enhance T-cell proliferation. Activation of the MAPK signaling pathway is a possible mechanism for the anti-apoptosis effect on the proliferation of CIK cells. In conclusion, anti-CD20 mAb may play an important role in the improvement of CIK-mediated cytotoxicity to tumor cells. These observations may aid in the improvement of the effects of immunotherapy in depleting the residual cells of hematopoietic tumors. Thus, the use of CIK cells cultured with anti-CD20 mAb could be a novel therapeutic strategy for the depletion of chemotherapy-resistant or residual cells in anaplastic large and B-cell lymphoma.

## Introduction

Residual tumor cells are a major complication in the therapy of hematopoietic tumors. The prognosis for patients with relapse due to the presence of residual tumor cells remains unsatisfactory (1). Therefore, the development of therapy for the depletion of these cells is important in the therapy of hematopoietic tumors.

Immunotherapy to stimulate the immune system of the patient is a promising method of depleting the residual cells of hematopoietic tumors. Cytokine-induced killer (CIK) cells are major histocompatibility complex (MHC)-unrestricted cytotoxic lymphocytes that can be induced by anti-CD3 monoclonal antibody (mAb), interleukin (IL)-2, IL-1 and interferon (IFN)-γ, include CD3^+^CD56^+^ cells and have high proliferation potential. The activity of CIK cells against tumor cells is an effective and MHC-unrestricted (2,3). CIK cells have the capacity to migrate toward tumor sites (4,5) and display antitumor activity *in vivo* (5). However, the clinical applicability of CIK cells to deplete residual leukemic cells has not been proven by various phase I studies performed thus far (6,7). The most relevant reason may be the limited basal antitumor activity of CIK cells. CIK cells exhibited a mean lytic activity of only 40% against the leukemic cells of patients in an *in vitro* assay (7). Therefore, it is necessary to increase the antitumor activity and the clinical applicability of CIK cells.

Rituximab is an anti-CD20 mAb used in the therapy of diffuse large B-cell lymphoma (DLBCL). In clinical trials, the use of rituximab alone or in combination with chemotherapy regimens as the first-line treatment has been shown to significantly improve response and survival for DLBCL (8–10).

In the present study, CD3^+^CD56^+^ cells were acquired from the peripheral blood of healthy donors and cultured *in vitro* in the presence of cytokines combined with rituximab to generate CIK cells. The antitumor activity of CIK cells to the SU-DHL2 and K562 human leukemia cell lines was investigated. A preliminary investigation to elucidate the mechanism was then performed.

## Materials and methods

### Human cell lines

One week prior to the experiment, the (SU-DHL2) cell line and the human chronic myelogenous leukemia cell line K562 (provided by the Cell Bank of the Shanghai Institute of Cell Biology, Chinese Academy of Science, Shanghai, China) were maintained in RPMI-1640 medium supplemented with 10% heat-inactivated fetal calf serum, 50 U/ml penicillin and 50 mg/ml streptomycin (Invitrogen Life Technologies, Carlsbad, CA, USA; further referred to as ‘complete medium’).

### Generation of CIK cells

Peripheral blood CD3^+^CD56^+^ cells were isolated by negative selection from 12 healthy donors from the laboratory and department and collected by venipuncture. Cells were isolated by negative selection from fresh blood using magnetic beads (CD3^+^CD56^+^ NKT Cell Isolation kit; Miltenyi Biotec, Bergisch Gladbach, Germany). Cells were cultured in complete medium at a density of 3×10^6^ cells/ml/well with recombinant human IFN-γ (1×10^6^ U/l), recombinant human IL-2 (rhIL-2; 5×10^5^ U/l; PeproTech Inc., Rocky Hill, NJ, USA), mouse anti-human CD3 monoclonal antibody (50 μg/l; Aibo Trading Co. Ltd, Shenzen, China) and clinical grade rituximab (5×10^4^ μg/l; Rituxan^®^; Roche, Basel, Switzerland) at 37°C with 5% CO_2_.

### Flow cytometry

Phenotypic analysis of the cells obtained from CIK cultures after washing twice with phosphate-buffered saline (PBS) was performed by mAb staining, using peridinin-chlorophyll-protein complex (PerCP)-anti-CD3, PerCP-anti-CD4, fluorescein isothiocyanate (FITC)-anti-CD56, FITC-anti-CD25, phycoerythrin (PE)-anti-perforin, PE-anti-granzyme B (Becton-Dickinson Biosciences, Franklin Lakes, NJ, USA) and PE-anti-CD314 (Beckman Coulter, Milan, Italy) on day 14. The cells (1×10^6^) were incubated with various conjugated mAbs for 30 min at room temperature, washed twice in PBS and then analyzed using a FACSCalibur^™^ flow cytometer (Becton-Dickinson Biosciences).

### Cytotoxicity assays

After 14 days in culture, rituximab was washed out of the experimental culture using PBS. Cytotoxicity of the CIK cultures against the SU-DHL2 and K562 cell lines were measured using the lactate dehydrogenase (LDH; Cytotoxicity Colorimetric Assay kit; Sigma-Aldrich, St. Louis, MO, USA) assay method. Effector (CIK) cell/target (SU-DHL2 or K562) cell (E/T) ratios of 10:1, 20:1 and 40:1 were used in the experimental and control groups. The SU-DHL2 or K562 cells of the two groups were seeded at a density of 2×10^5^ cells/ml in round-bottomed 96-well plates (Nunc A/S, Roskilde, Denmark) in RPMI-1640 complete medium (in a final volume of 200 ml/well) at 37°C, 5% CO_2_ for 72 h. All the groups were seeded three times concurrently. The plates were then centrifuged for 1 min at 10,000 × g. Then, 100 μl supernatant was collected from each well and transferred to a new flat-bottomed 96-well plate. Subsequent to the effects of the reaction and stop solutions, the absorbance was measured at the recommended wavelength using a Multiskan MS ELISA reader (Bio-Tek, Winooski, VT, USA).

### Reverse transcription-quantitative polymerase chain reaction (RT-qPCR)

After 14 days in culture, rituximab was washed out of the experimental culture using PBS. Cells in the experimental and control groups were harvested and used for qPCR analysis. Total RNA extracted from the cells with TRIzol^®^ reagent (Invitrogen Life Sciences) was used as the template for all reverse transcriptase reactions. The cDNA was synthesized with random priming from 10 μl total RNA with the aid of the Revert Aid™ First Strand cDNA Synthesis kit (Fermentas, Carlsbad, CA, USA), following the manufacturer’s instructions. For the PCR, 2 μl cDNA solution was mixed with 10 μl SYBR^®^ Premix Ex Taq II (Takara Bio Inc., Shiga, Japan), 0.4 μl each of the forward and reverse primers and 7.2 μl RNase-free water to provide a total volume of 20 μl. A fluorescent quantitation PCR cycler (LightCycler^®^; Roche) was used for amplification of perforin, granzyme B, INF-γ, tumor necrosis factor (TNF)-α, TNF-β and Fas ligand (FasL) with the primer pairs listed in Table I. The amplification consisted of denaturation at 95°C for 5 sec, annealing at 62°C for 20 sec and extension at 72°C for 10 sec (43 cycles). The threshold cycle (Ct) was subsequently determined. Expression levels of perforin, granzyme B, INF-γ, TNF-α, TNF-β and FasL, normalized to GAPDH and relative to a calibrator, was expressed as 2^−ΔΔCt^ (fold difference). All procedures were performed following the manufacturer’s instructions.

### Western blot analysis

After 14 days in culture, rituximab was washed out of the experimental culture using PBS. Cells in the experimental and control groups were harvested and used for western blot analysis to detect the constitutively activated signal transduction pathways in the CIK cells. Total protein was separated by SDS-PAGE and then electrotransferred to a polyvinylidene fluoride membrane, using a transfer apparatus set to 100 V for 1 h at 4°C. After washing, the membranes were probed with the following primary antibodies: signal transducer and activator of transcription 1 (STAT-1), STAT-3, STAT-5 mouse anti-rat monoclonal antibodies (1:1,000; Millipore, Billerica, MA, USA), extracellular signal-regulated kinase (ERK) 1/2 activated mouse anti-rat monoclonal antibody and p38 mitogen-activated protein kinase (MAPK) mouse anti-rat polyclonal antibody (1:1,000; Cell signaling Technology, Inc., Danvers, MA, USA). After the membranes had been agitated gently for at least 1 h, the antibody solution was poured off the membrane and the membrane was washed twice for 10 min with Tris-buffered saline and Tween 20 (TTBS) buffer. The mouse anti-human monoclonal secondary antibody (Santa Cruz, Dallas, TX, USA) was added at a dilution of 1:5,000 in 5 ml 0.5% blocking buffer. The secondary antibody solution was then poured off the membrane, which was subsequently washed twice. The TTBS buffer was poured off and the developing reagent added. When the bands were clearly observed, development was stopped by washing the membrane with distilled water for 30 min with three changes.

### Statistical analysis

All data are expressed as the mean ± standard deviation. Statistical analyses were performed with SPSS software, version 12.0 (SPSS, Inc., Chicago, IL, USA). Differences between repeated experiments of the two groups were investigated using the unpaired Student’s t-test and F-test. For all statistical tests, values of P<0.05 were considered as statistically significant.

## Results

### Cytotoxic activity of CIK cells

**Figure 1:**
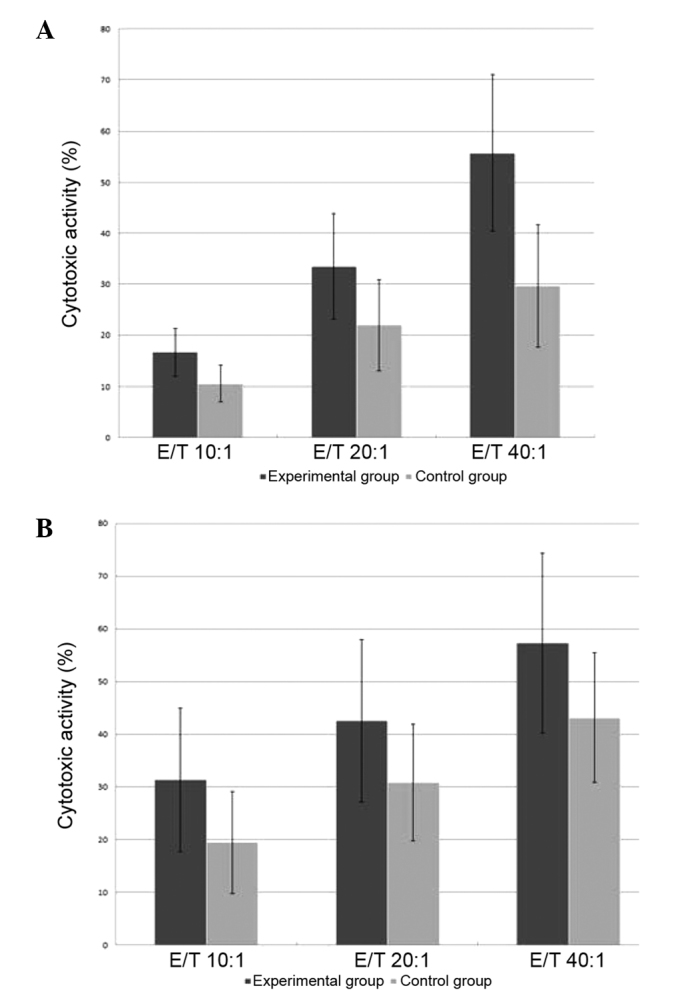
Cytotoxicities of CIK cells against (A) SU-DHL2 cells at the different ratios of E/T (10:1, 20:1 and 40:1) in the experimental (rituximab) group (16.64±4.75, 33.53±10.36 and 55.73±15.33%, respectively) were higher than those in the control group (10.54±3.55, 21.96±8.94 and 29.64±12.05%, respectively; P<0.05). Cytotoxicities of CIK cells against (B) K562 cells at the different ratios of E/T in the experimental group (31.32±13.59, 42.58±15.37 and 57.27±17.08%, respectively) were higher than those in the control group (19.47±9.69, 30.85±11.06 and 43.12±12.31%, respectively; P<0.05). CIK, cytokine-induced killer; E/T, effector cell/target cell ratio.

The cytotoxicity of the day-14 CIK cells against the SU-DHL2 and K562 cell lines was measured using the LDH assay method. The cytotoxic activity with different E/T ratios was determined in the two groups against the two cell lines. Cytotoxicities against the SU-DHL4 or K562 cells at each of the different E/T ratios in the experimental (rituximab) group were higher than those of the control group (P<0.05; Fig. 1).

### Expansion, phenotype and cytokine secretion analysis of CIK cells

**Figure 2:**
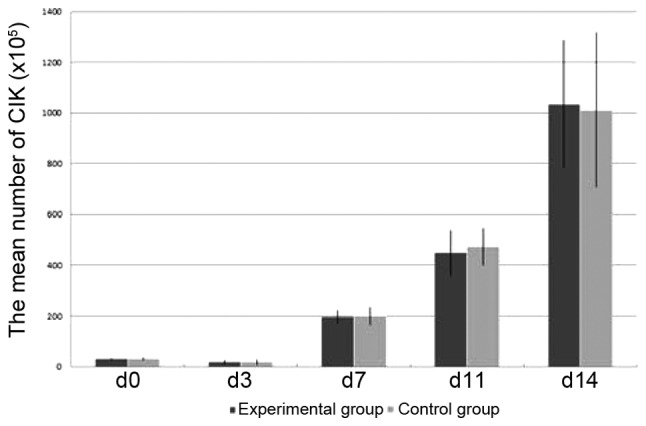
The mean number of cells in the experimental (rituximab) group increased from (31.1±3.6)x10^5^ to (1,035.1±251.4)x10^5^, thus representing a mean 33.29-fold expansion. The mean number of cells in the control group increased from (30.7±5.1)x10^5^ to (1,011.8±305.1)x10^5^, thus representing a mean 32.53-fold expansion (P>0.05).

Starting from (31.1±3.6)x10^5^ cells in the experimental group and (30.7±5.1)x10^5^ cells in the control group, the cells of the two groups were counted to determine the absolute cell number on days 3, 7, 11 and 14 and were harvested on day 14. After 14 days of expansion, the mean total number of experimental group cells was ~(1,035.1±251.4)x10^5^, thus representing a mean 33.29-fold expansion. The mean total number of the control group cells was ~(1,011.8±305.1)x10^5^, thus representing a mean 32.53-fold expansion (Fig. 2). No significant difference was identified between the mean cell counts of the two groups (P>0.05).

**Figure 3:**
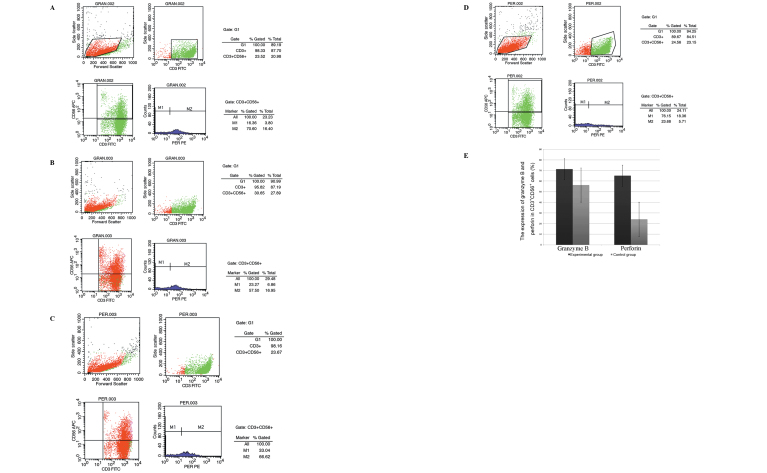
The mean numbers of granzyme B-positive cells and perforin-positive cells expressed in CD3^+^CD56^+^ cells in the experimental (rituximab) group were higher than those in the control group (P<0.05). (A) Granzyme B (experimental group; 71.25±21.65%); (B) granzyme B (control group; 56.29±16.99%); and (C) perforin (experimental group; 65.08±17.47%). FITC, fluorescein isothiocyanate; PE, phycoerythrin. The mean numbers of granzyme B positive cells and perforin positive cells expressed in CD3^+^CD56^+^ cells in the experimental (rituximab) group were higher than those in the control group (P<0.05). (D) Perforin (control group; 20.05±6.97%); (E) the mean percentages of granzyme B and perforin expressed in CD3^+^CD56^+^ cells. FITC, fluorescein isothiocyanate; PE, phycoerythrin.

The CD3^+^CD56^+^ cells in the two groups were analyzed on day 14. The final population comprised a mean of 93.37±17.48% CD3^+^CD56^+^ cells in the experimental group and 94.15±16.96% in the control group. The expression of granzyme B and perforin in the CD3^+^CD56^+^ cells on day 14 was analyzed. The final CD3^+^CD56^+^ cell population in the experimental group comprised a mean of 71.25±21.65% granzyme B-positive cells and 65.08±17.47% perforin-positive cells. However, in the control group the final CD3^+^CD56^+^ cell population comprised a mean 56.29±16.99% granzyme B-positive cells and 20.05±6.97% perforin-positive cells. The mean proportions of granzyme B- and perforin-positive cells among the CD3^+^CD56^+^ cells in the experimental group were higher than those in the control group at the end of the expansion period (P<0.05; Fig. 3). The experiment was repeated three times with similar results.

**Figure 4:**
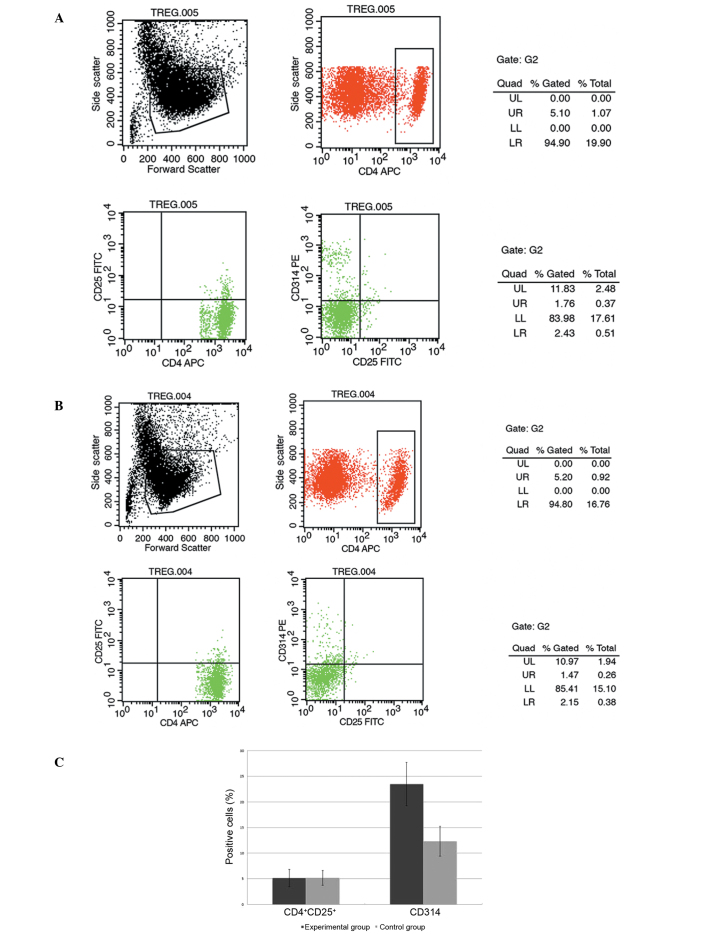
The mean number of CD314^+^ cells expressed in the experimental (rituximab) group was higher than that in the control group (p<0.05). No difference was identified between the mean number of CD4^+^CD25^+^ cells expressed in the experimental (5.16±1.68%) and control groups (5.19±1.43%) (P>0.05). CD314^+^ cells expressed in the (A) experimental (23.49±4.22%) and (B) control groups (12.35±2.94%) on day 14; (C) the mean percentages of CD4^+^CD25^+^ and CD314^+^ cells. FITC, fluorescein isothiocyanate.

The CD4^+^CD25^+^ and CD314^+^ cells were analyzed on day 14. The final population in the experimental group comprised a mean of 5.16±1.68% CD4^+^CD25^+^ cells and 23.49±4.22% CD314^+^ cells. However, in the control group the final population comprised a mean of 5.19±1.43% CD4^+^CD25^+^ cells and 12.35±2.94% CD314^+^ cells. The mean proportion of CD314^+^ cells in the experimental group was higher than that in the control group at the end of the expansion period (P<0.05; Fig. 4). No difference was identified between the mean proportions of CD4^+^CD25^+^ cells observed in the two groups (P>0.05). The experiment was repeated three times with similar results.

### RT-qPCR analysis

**Figure 5:**
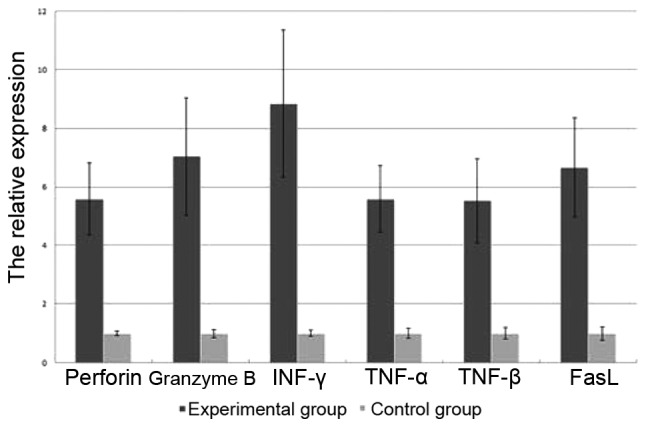
Expression levels of perforin, granzyme B, INF-γ, TNF-α, TNF-β and FasL in the experimental group were higher than those in the control group (P<0.05). INF, interferon; TNF, tumor necrosis factor; FasL, Fas ligand.

The relative expression levels of perforin, granzyme B, INF-γ, TNF-α, TNF-β and FasL of the two groups were detected by RT-qPCR analysis on day 14. After 14 days of expansion, the expression levels of perforin, granzyme B, INF-γ, TNF-α, TNF-β and FasL in the experimental group were significantly higher than those in the control group (P<0.05; Fig. 5).

### Signaling pathway analysis by western blotting

**Figure 6:**
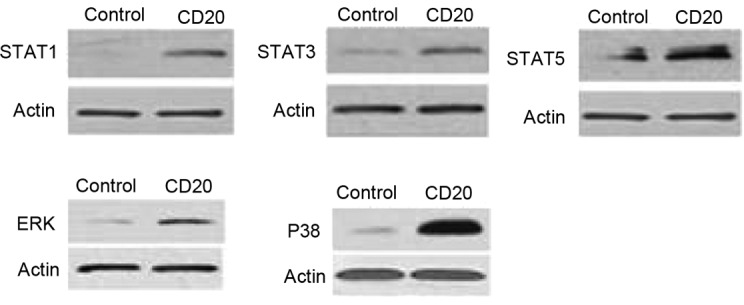
Expression levels of STAT1, STAT3, STAT5, ERK 1/2 and p38 mitogen-activated protein kinase in the experimental group were higher than those in the control group. STAT, signal transducer and activator of transcription; ERK, extracellular signal-regulated kinase.

In this study, the effects of rituximab on the STAT-1, STAT-3, STAT-5, ERK 1/2 and p38 MAPK signaling pathways of CIK cells associated with proliferation and apoptosis regulation were analyzed. The results indicate that components of the STAT-1, STAT-3, STAT-5, ERK 1/2 and p38 MAPK signal transduction pathways were constitutively phosphorylated in the rituximab group compared with the control group (Fig. 6).

## Discussion

Residual tumor cells represent a major obstacle in the therapy of malignant hematological disease including acute leukemia (AL) and lymphoma. The prognosis for patients that relapse remains unsatisfactory. Therefore, the development of therapy with regard to the depletion of residual tumor cells is important for the treatment of such diseases. CD3^+^CD56^+^ cells can be generated from healthy donors. CIK cells have exhibited anticancer activity *in vitro* and *in vivo* (11,12). The cytotoxicity to tumor cells is non-MHC-restricted, but relies on cell-to-cell contact, and is perforin-dependent and Fas-independent (13). Additionally, CIK cells express NKG2D (CD314) and perforin, two molecules that have been demonstrated to play major roles in CIK-mediated cytotoxicity (14). Another feature of CIK cells is the production of effector cytokines including IFN-γ, TNF-α, IL-2 and IL-12, which are involved in the immunoregulation in anticancer activity. CIK cells exhibit activity against leukemia targets while having low or absent activity against normal bone marrow stem cells and tissues (15). In the present study, CIK cells were generated from the peripheral blood of healthy donors by exposure to rituximab (an anti-CD20 mAb) in the culture process. The results indicated that rituximab increased the cytotoxicity of the CIK cells against SU-DHL2 and K562 cells. Furthermore, the present study may provide a novel useful method for the cultivation of CIK cells and depletion of residual cells for AL and lymphoma patients.

Rituximab as an anti-CD20 mAb is a main treatment used in the therapy of a broad variety of B-cell malignancies. Rituximab alone or as an addition to chemotherapy can enhance the complete response, long-term remission and cure rate (9). The mechanisms responsible for the antitumor effects of rituximab are not fully understood. However, direct signaling, direct induction of apoptosis, complement-dependent cellular cytotoxicity and antibody-dependent cellular cytotoxicity (ADCC) are potential mechanisms of action of rituximab.

In the present study rituximab was added to the CIK cultures. Although no difference was identified in the expansion in the experimental and control groups, the cytotoxicity in the experimental group against the SU-DHL2 and K562 cell lines was increased. A previous study demonstrated that the addition of anti-CD20 mAb increased the killing activity of CIK cells toward B-cell non-Hodgkin’s lymphoma (B-NHL) cell lines (16). This enhancement was mainly due to ADCC mediated by the 1–10% natural killer cells that contaminated the CIK cultures. These data suggest that an anti-CD20 mAb could be used *in vivo* to enhance CIK therapeutic activity in B-NHL cell lines.

In the present study, certain other features of the increased cytotoxicity of CIK cells induced by the anti-CD20 mAb were demonstrated. It was observed that the proliferation of the CIK cells was not increased by the addition of anti-CD20 mAb; however, the relative expression levels of perforin, granzyme B, INF-γ, TNF-α, TNF-β and FasL detected by RT-qPCR analysis were significantly enhanced. These effector cytokines are produced by CIK cells and are involved in the immunoregulation in anticancer activity. The results revealed that the expression of the CIK cell phenotype CD3^+^CD56^+^ was not improved by the addition of anti-CD20 mAb. However, the proportions of granzyme B, perforin and CD314^+^ cells in the CD3^+^CD56^+^ cells analyzed by flow cytometry were significantly enhanced. The increase of CIK cell cytotoxicity is associated with the increase in the expression of the proposed cytotoxic factors. Therefore, anti-CD20 mAb could play an important role in the improvement of the cytotoxicity of CIK cells by enhancing these cytotoxic factors.

The present study demonstrated that in the CIK cells, the anti-CD20 mAb promoted the STAT and MAPK/ERK signaling pathways that play central roles in cell growth and apoptosis. STAT1 is a well-characterized component of the IFN-induced signaling pathways (17). STAT1 is directly associated with T-cell apoptosis and may also contribute to the events that govern the elimination of autoreactive T cells via negative selection (18). STAT1, STAT3 and STAT5 are important co-stimulatory molecules that lead to the enhancement of T-cell proliferation (19). STAT5 is an important component downstream of cytokines that regulate T-cell biology and a critical survival factor in T-cell development and survival (20,21). The present study reveals a critical role for the anti-CD20 mAb in T-cell development and survival by the upregulation of the expression of STAT proteins. It was also demonstrated that the expression of ERK 1/2 and p38 increased due to the effects of the anti-CD20 mAb. MAPK consists of ERK 1/2, c-Jun N-terminal kinases, p38 and ERK 5/Big MAPK family members (22). The increased basal and activated p38 MAPK signaling pathway is critical to death receptor resistance (23). Due to interaction between the p38 MAPK pathway and TNF-nuclear factor κB signaling, the role of p38 in acquired apoptosis resistance is of biological and therapeutic interest (24). The apoptosis-resistance effect of the activated MAPK signaling pathway is a possible mechanism underlying the anti-apoptosis effect of anti-CD20 mAb on the proliferating CIK cells.

The data suggest that rituximab, an anti-CD20 mAb, could be used to enhance the antitumor activity of CIK cells against SU-DHL2 and K562 cell lines. The increase of CIK cell cytotoxicity derived from the addition of anti-CD20 mAb to the CIK cell culture was found to be associated with an increase in the expression of cytotoxic factors. The results also revealed that the effects of the anti-CD20 mAb were associated with an increase in the expression of components of the STAT and MAPK/ERK signaling pathways. Upregulation of the expression of STAT1, STAT3 and STAT5 is important as these co-stimulatory molecules enhance T-cell proliferation, and the activated MAPK signaling pathway is a possible mechanism underlying the anti-apoptosis effect on the proliferating CIK cells. The anti-CD20 mAb was demonstrated to improve CIK-mediated cytotoxicity to SU-DHL2 or K562 cell lines. In conclusion, CIK cells cultured with anti-CD20 mAb could be a novel therapeutic strategy for the depletion of chemotherapy-resistant or residual cells of AL and B-cell lymphoma.

## Figures and Tables

**Table I:** List of primers used for the quantitative polymerase chain reaction analysis.

Target gene	Primer sequences	Amplification length (bp)
Perforin	Forward 5′-CAGGTCAACATAGGCATCCACG-3′ Reverse 5′-GAACAGCAGGTCGTTAATGGAG-3′	160
Granzyme B	Forward 5′-GAAACGCTACTAACTACAGG-3′ Reverse 5′-CCACTCAGCTAAGAGGT-3′	126
INF-γ	Forward 5′-GCAGAGCCAAATTGTCTCCT-3′ Reverse 5′-ATGCTCTTCGACCTCGAAAC-3′	290
TNF-α	Forward 5′-CGAGTGACAAGCCTGTAGC-3′ Reverse 5′-CCTTCTCCAGCTGGAAGAC-3′	363
TNF-β	Forward 5′-AGGCATGAGGGATCACAG-3′ Reverse 5′-AAAGAGGTTTATTGGGCTTC-3′	115
FasL	Forward 5′-TGTTTATGAGCCAGACAAATGG-3′ Reverse 5′-AAGACAGTCCCCCTTGAGGT-3′	203

INF, interferon; TNF, tumor necrosis factor; FasL, Fas ligand.
